# Reduced fetal movements and COVID-19 infection: a retrospective cohort study

**DOI:** 10.1186/s12884-025-07196-w

**Published:** 2025-01-30

**Authors:** Amira Gentili, Irene Sterpu, Joanna Tingström, Eva Wiberg-Itzel

**Affiliations:** 1 Department of Clinical Science and Education, Department of Obstetrics and Gynecology, Karolinska Institute, Sodersjukhuset, Stockholm, 118 83 Sweden; 2 Division of Obstetrics and Gynecology, Department of Clinical Sciences, Intervention and Technology (CLINTEC), Karolinska Institutet, Stockholm, Sweden

**Keywords:** Reduced fetal movements, Intrauterine fetal death, Stillbirths, COVID-19

## Abstract

**Background:**

Fetal movements are an important indicator of fetal well-being; therefore, reduced fetal movements (RFMs) can indicate fetal compromise. RFM is associated with fetal growth restriction (FGR) and intrauterine fetal death (IUFD). Studies have implied that COVID-19 infection increases the risk of adverse fetal outcomes, such as preterm birth and IUFD. It is unclear how COVID-19 infection may aggravate these fetal outcomes among women presenting with RFM.

The aims of the study were to (1) determine whether adverse fetal outcomes in women with RFM increased in 2020 compared to 2019, the year before the pandemic, and (2) evaluate whether maternal COVID-19 infection during pregnancy was a risk factor for adverse fetal outcomes in comparison to previously established risk factors among women seeking care for RFM.

**Methods:**

All women who sought care due to RFM and were delivered at Soder Hospital from 2019 to 2020 were included. Fetal composite outcomes were constructed and compared between women with RFM and COVID-19 and women with RFM but without COVID-19.

**Results:**

COVID-19 infection did not increase the risk of adverse fetal outcomes in women who sought care for RFM. A twofold risk for adverse fetal outcomes was found among all primiparous women vs. multiparous women with RFM (98/788 [12.4%] vs 37/644 [9.8%], AOR = 2.5, 95% CI (1.6–3.7).

**Conclusion:**

The proportion of adverse composite outcomes among women with RFM during the first year of the pandemic did not increase compared to the year before. Composite outcomes were marginally higher in the COVID-19-positive group compared to the COVID-19-negative group, but it was not statistically significant.

## Introduction

Pregnancy was identified as an essential risk factor for severe COVID-19 disease during the pandemic [1, 2]. Reduced fetal movement (RFM) during pregnancy is a common reason for antenatal visits and is associated with the risk of adverse pregnancy outcomes, the most serious of which is intrauterine fetal death (IUFD) [3, 4]. It is hitherto unclear whether there is an association between increased adverse fetal outcomes and pregnant women’s experience of RFM during a COVID-19 infection.

Fetal movement is defined as the “physical activity of the fetus in utero” [5]. It is described as the maternal sensation of any kick, swish or flutter the fetus performs. These movements are essential for the development of the neurological, muscular, and skeletal systems of the fetus [6].

Fetal growth restriction (FGR) is diagnosed when growth, for various causes, is suboptimal. FGR increases the risk of adverse fetal outcomes [7], such as IUFD [8, 9], and research has identified a relationship between RFM and FGR.

In the Swedish context, IUFD is defined as the birth of a stillborn baby after 22 weeks of gestation [10]. The incidence of stillbirth in Sweden has remained relatively constant for the last 30 years [7], with 367 deaths in 2019 (3.2 per 1,000 born) and 353 deaths in 2020 (3,12 per 1000 born), the year before the pandemic. The causes of IUFD can be divided into three major categories: FGR due to placental insufficiency, congenital malformation of the fetus and unknown causes [11, 12]. Moreover, infection is linked to IUFD and accounts for approximately 6–15% of all IUFD cases [12]. As mentioned above, the levels of IUFD in Sweden have remained relatively constant in the last 30 years. However, it is unclear whether maternal COVID-19 infection impacts IUFD rates. While a study conducted in the United Kingdom found no impact of COVID-19 infection on IUFD rates, it found a significant decrease in attendance for the first episode of RFM during the pandemic compared to pre-pandemic times [13].

The current understanding of the impact of COVID-19 on fetal outcomes is multifaceted. Studies have shown that COVID-19 infection can cause several adverse fetal outcomes, such as preterm birth and IUFD [2, 14, 15]. However, other studies have failed to observe an increase in IUFD as an adverse fetal outcome during the COVID-19 pandemic [16, 17]. A Swedish study found no association between being born during the early period of the pandemic and preterm births and IUFDs compared to pre-pandemic times [18]. The results so far have been predominantly from studies conducted outside of Scandinavia and have not considered the direct effect of the association of maternal COVID-19 infection and RFM on fetal outcomes.

## Materials and method

### Aims of the study

This study sought to determine whether adverse fetal outcomes in women with RFM increased in 2020 compared to the year before the pandemic (2019). Second, the objective was to evaluate whether maternal COVID-19 infection during pregnancy was a risk factor for adverse fetal outcomes compared to previously established risk factors among women seeking care for RFM.

### Ethical approval

Ethical permission was obtained (diary number 2021–00480). The study complied with the World Medical Association Declaration of Helsinki regarding the ethical conduct of research involving human subjects.

### Study design

The study employed a retrospective cohort design based on medical files from January 2019 to December 2020. Data were collected from the medical records system Obstetrix (Obstetrix Cerner, Sverige AB). All deliveries occurred at Soder Hospital in Stockholm, one of Sweden’s largest birth suites. All the women included in the study had sought care for RFM at least once during pregnancy and delivered at the hospital during the study period. In this study, RFM was defined as existing according to the pregnant woman’s subjective experiences and was registered based on an International Disease Classification code for RFM.

### Study population

The criteria for inclusion in the COVID-19-infected group were women who tested positive for COVID-19 at their clinic visit, either in outpatient care or in the birth suite, through a polymerase chain reaction (PCR) test. The test was performed in outpatient care when the pregnant woman showed symptoms of infection (non-universal testing). All women who attended the labour ward for childbirth were tested (universal testing). Self-reported cases of COVID-19 and home tests were not included in the study. There was no requirement for a specific time frame for detecting the virus with the episodes of RFM. Women with a history of RFM and who had never delivered at Soder Hospital were excluded from the study.

The study outcome was to compare the number of women who sought care for RFM in 2019 and 2020. Second, a composite of adverse fetal outcomes was created and registered for all pregnancies with RFM if one or more of the following criteria were met: 1) pH < 7.10 in the umbilical artery at birth; 2) a five-minute Apgar score < 7; 3) the need for resuscitation resulting from fetal distress (heart massage and/or intubation); 4) admission to the neonatal intensive care unit (NICU) for more than 24 h and 5) IUFD from the 22nd week.

In this study, we analysed risk factors that increased the likelihood of an adverse fetal outcome [7]. The evaluated risk factors included primiparity, advanced maternal age (above 35), body mass index (BMI > 30), smoking before pregnancy, chronic illnesses before pregnancy, pregnancy-related illnesses, more than one visit for RFM, gestational age, and small for gestational age (SGA) and large for gestational age (LGA) measurements.

All the background data – such as maternal body mass index (BMI), parity, age, previous caesarean section, past illnesses, pregnancy complications and fetal data such as SGA or LGA – were collected from the medical records of the pregnant women. Data on pregnancy outcomes were also obtained from these records. The birth charts also provided information about the newborns, such as their gender, birth weight, length, pH in the umbilical artery, Apgar scores at birth, resuscitation, including heart massage and intubation, and admission to the NICU.

### Statistical analysis

SPSS Statistics (version 28, IBM Corp., Armonk, NY, USA) was used for the statistical analyses. Descriptive analyses were performed on the data from all the pregnancies included in the study. To demonstrate differences between the continuous variables when comparing maternal and fetal background data – such as age, BMI, weight, length, and head measurements – a two-sided independent sample t-test was used. Wald Chi-Squared statistics were used for categorical data such as parity, smoking, IVF, etc. The result was presented as proportions (%) and/or averages, with standard deviations (SD) as the spread measure. The significance level was decided at a *p*-value of < 0.05. A bivariate regression analysis was performed to identify associations between risk factors and adverse fetal outcomes. These were presented with an unadjusted odds ratio (OR) and an adjusted odds ratio (AOR), together with a 95% confidence interval (CI). Furthermore, the Hosmer–Lemeshow model of goodness of fit was used to examine whether the adjusted model adequately fit the data, with *p* > = 0.05 indicating a good fit.

## Results

**Fig. 1:**
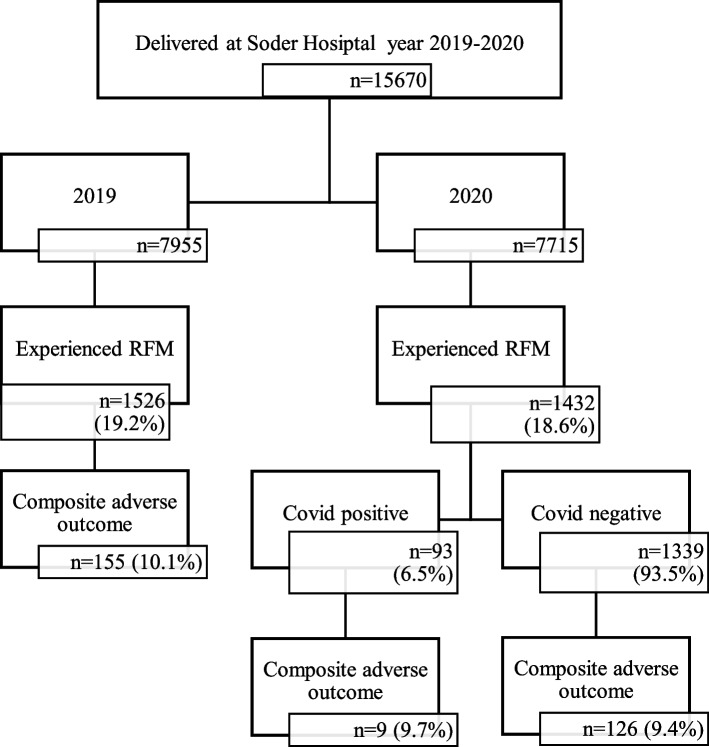
A total of 15,670 deliveries occurred at Soder Hospital during the study time, among them 2958 experienced RFM and were included in the study

During 2019 and 2020, 2,958 women sought care for RFMs at Soder Hospital in Stockholm (Fig. 1). These women made 3,761 visits due to RFM. The number of visitors in each year differed, with a slight decrease in the first year of the pandemic compared to pre-pandemic times (19.2% vs 18.6%).

Table 1 presents information on the maternal background and labour characteristics, divided according to the year of visit at the clinic.

**Table 1:** Baseline data for women with RFM in 2019 and 2020. Deliveries are divided into groups according to the year of visit. Values are presented as numbers (%) or means (± SD)

Total *n* = 2,958	2019 *n* = 1,526	2020 *n* = 1,432	*p*-value
Maternal characteristics			
Age (years)	32 (± 5)	33 (± 5)	0.49
Parity (% of primiparas) Missing: 50	853 (57.8)	788 (55.0)	0.13
BMI (kg/m2) Missing 207	24.5 (± 4.2)	24.7 (± 4.4)	0.24
Smoking before pregnancy A (%) Missing: 220	208 (14.8)	144 (10.8)	0.002^*^
Chronic illness before pregnancyB (%) Missing: 132	174 (12.0)	176 (12.8)	0.48
COVID-19 infectionC	0 (0.0)	93 (6.5)	< 0.001^*^
Pregnancy-related illnessD	173 (11.3)	155 (10.8)	0.65
Number of RFM visits	1.28 (± 0.6)	1.26 (± 0.6)	0.27
Onset of labour			
Induction of labour (%)	374 (24.5)	414 (28.9)	0.007^*^
Spontaneous onset of labour (%)	985 (64.5)	827 (57.8)	< 0.001^*^
Elective caesarean (%)	158 (10.4)	174 (12.2)	0.12
Manner of birth			
Spontaneous vaginal (%)	1140 (74.17)	1042 (72.8)	0.23
Vacuum/forceps (%)	89 (5.8)	94 (6.6)	0.40
Emergency caesarean (%)	139 (9.1)	118 (8.2)	0.40
Time of stay at the hospital (hours)	73.51 (± 38.26)	66.52 (± 38.06)	< 0.001^*^
Pelvic floor injuries (%) Grade 3 + 4	44 (2.9)	36 (2.5)	0.53
Infection post-birth Yes (%)	33 (2.6)	16 (1.4)	0.047^*^
Post-partum haemorrhage > 1.0 L Yes (%)	145 (10.5)	139 (10.6)	0.94

A: Smoking before three months of pregnancy. B: Diabetes mellitus, cardiovascular disease, hypertension, lung disease/asthma or chronic kidney disease. C: Tested on arrival at the clinic before labour and/or when seeking care for RFM. D: Preclampsia, hypertension, gestational diabetes, hepatopulmonary syndrome or gestational diabetes

A: Pelvic floor injuries, Grades 3 and 4 only

^*^Considered statistically significant if *p* < 0.05

In 2019, 19% (*n* = 1,526) of all pregnant women who delivered at the clinic sought care for RFM. A similar number was recorded in 2020: 18% (*n* = 1,432) of all pregnancies. The Mean age of the women in the study was 32 years (17–53). The frequency of women who smoked before pregnancy was significantly higher in 2019 compared to 2020 (14.5% vs 10.8%; *p* = 0.002).

Among the women who sought care for RFM during the study period, the average number of visits was 1.3 times during pregnancy. No difference was shown between the years (1.28 vs. 1.26 visits, *p* = 0.27). In 2020, there was a higher rate of labour induction (28.9%) and a decrease in the number of spontaneous labour onsets (57.8%) compared to 2019 (24.5%, respectively, 65.5%).

In Table 2, birth data was presented.

**Table 2:** Fetal data and outcomes in 2019 and 2020. Deliveries data presented per year. Values are presented as numbers (%) or means (± SD)

Total *n* = 2,958	2019 *N* = 1,526	2020 *n* = 1,432	*p*-value
Fetal characteristics			
Gestational age (days) Missing: 82	279 (± 12)	279 (± 11)	0.71
Gender, boys (%) Missing: 2	754 (49.4)	698 (48.7)	0.32
Fetal weight (g) Missing: 1	3.511 (± 513.7)	3.512 (± 509.3)	0.98
Fetal length (cm) Missing: 8	50.12 (± 2.1)	50.03 (± 2.2)	0.28
Fetal head circumference (cm) Missing: 22	35.01 (± 2.0)	35.12 (± 1.7)	0.11
At birth			
^a^Apgar score < 7 at 5 min after birth Yes (%)	18 (1.20)	12 (0.8)	0.35
^a^PH < 7.10 (%)	50 (4.0)	57 (4.8)	0.33
^a^IUFD (%)	3 (0.2)	2 (0.1)	0.70
^a^Need for resuscitation Yes (%)	1 (0.1)	2 (0.1)	0.52
^a^Transfer to NICU Yes (%)	91 (6.0)	77 (5.4)	0.49
Composite outcomes (%)	155 (10.2)	135 (9.4)	0.50

*Abbreviations*: *IUFD* intrauterine fetal death, *NICU* neonatal intensive care unit

^a^composite adverse neonatal outcomes: 1) pH less than 7.10 in the umbilical artery; 2) 5-min Apgar score less than 7; 3) the need for resuscitation (heart massage and/or intubation) resulting from fetal distress; 4) admission to the neonatal intensive care unit for more than 24 h; 5) IUFD; or 6) all of these. Some of the neonates who were classified as having adverse neonatal outcomes had more than one of the factors identified

The results from the fetal data and outcomes show that 290 (9.8%) of the newborns in the population had one or more of the factors relating to the composite outcomes identified, five of which were due to IUFD (*p* = 0.7). Adverse fetal outcomes were recorded for 10.2% of the newborns in 2019 compared to 9.4% in 2020 (*p* = 0.5). No differences in the gestational age of the newborns were identified between the two years (*p* = 0.71).

Table 3 presents the maternal characteristics for the two groups of women with RFM in 2020: those with COVID-19 (6.5% of all women, *n* = 93) and those without (*n* = 1,339).

**Table 3:** Women with reduced fetal movements (RFM) in 2020 were divided into groups according to those with COVID-19 (*n* = 93) or without (*n* = 1,339). Values are presented as numbers (%) or means (± SD)

Total *n* = 1,432	RFM with COVID-19 *n* = 93	RFM without COVID-19 *n* = 1,339	*p*-value
Maternal characteristics			
Age (years)	33.56 (± 5.1)	32.45 (± 4.5)	0.03^*^
Parity (% of primiparas)	51 (54.8)	737 (55.0)	0.97
BMI (kg/m2) Missing: 84	25.07 (± 4.2)	24.65 (± 4.4)	0.40
Smoking before pregnancyA (%) Missing: 98	15 (17.9)	129 (10.3)	0.03^*^
Chronic illness before pregnancy (%) Missing: 60	18 (20.7)	158 (12.3)	0.02^*^
Pregnancy-related illnessC	14 (15.1)	141 (10.5)	0.17
Number of RFM visits	1.41 (± 0.7)	1.25 (± 0.6)	0.02^*^
Onset of labour			
Induction	22 (23.7)	392 (29.3)	0.24
Spontaneous	42 (45.2)	785 (58.6)	0.01^*^
Elective caesarean	27 (29.0)	147 (11.0)	< 0.001^*^
Emergency caesarean	2 (2.2)	15 (1.1)	0.37
Manner of birth			
Spontaneous vaginal	55 (59.1)	987 (73.7)	0.002^*^
Vacuum/forceps	4 (4.3)	90 (6.7)	0.36
Emergency caesarean	7 (7.5)	111 (8.3)	0.79
Time of stay at the hospital (hours)	84.63 (± 63.78)	65.26 (± 35.30)	< 0.001^*^
Pelvic floor injuries (%)A			
Grades 3 and 4	4 (4.3)	32 (2.4)	0.25
Infection post-birth Yes (%) Missing: 309	3 (4.1)	13 (1.2)	0.05^*^
Post-partum haemorrhage > 1.0 L Yes (%) Missing: 124	12 (14.8)	127 (10.4)	0.20

A: Smoking before three months of pregnancy. B: Diabetes mellitus, cardiovascular disease, hypertension, lung disease/asthma or chronic kidney disease. C: Tested on arrival at the clinic before labour and/or when seeking care for RFM. D: Preclampsia, hypertension, gestational diabetes, hepatopulmonary syndrome or gestational diabetes

A: Pelvic floor injuries, Grade 3 and 4 only

^*^Considered statistically significant if *p* < 0.05

The mean number of RFM visits was higher in women with COVID-19 (1.41) than those without (1.25, *p* = 0.02). The COVID-19 group had a higher mean age (*p* = 0.03), higher smoking prevalence (*p* = 0.03) and higher chronic illness prevalence (*p* = 0.02) before pregnancy. Pregnant women with COVID-19 had higher rates of elective c-Sect. (29 vs. 11%) and lower rates of spontaneous labour (45.2 vs. 58.6%) compared to those without (11 vs 58.6%, *p* < 0.001). They also had higher rates of assisted birth (59.1%) and more extended hospital stays (84 h) compared to pregnant women without COVID-19 (73.7 h; and 65 h; *p* < 0.001).

The 2020 fetal data distribution (Table 4) revealed a lower gestational age among infants born to mothers with COVID-19 and RFM than those without COVID-19 (277 days vs 279 days, *p* = 0.05). Although the adverse composite outcomes were marginally higher in the COVID-19-positive group (9.7%) compared to the COVID-19-negative group (9.4%), they were not statistically significant. In 2020, there were two incidents of IUFD in the group of mothers without COVID-19. Of the total of 36 SGA babies, 35 were from mothers who did not have COVID-19. A higher percentage of infants admitted to the NICU were born to mothers in the COVID-19-positive group, although the difference was not statistically significant (6.5 vs. 5.3%).

**Table 4:** Fetal data and outcomes 2020. Deliveries during 2020 are divided into groups according to whether the mother contracted a COVID-19 infection during pregnancy. Values are presented as numbers (%) or means (± SD)

Total *n* = 1,432	RFM with COVID-19 *n* = 93	RFMwithout COVID-19 *n* = 1,339	*p*-value
Infant characteristics			
Gestational age (days) Missing: 32	277 (± 12)	279 (± 11)	0.05^*^
Gender, boys (%) Missing: 2	48 (51.6)	650 (48.6)	0.57
Fetal weight (g) Missing: 1	3,487(± 501)	3,514 (± 510)	0.62
Fetal length (cm) Missing: 3	49.9 (± 2.2)	50.1 (± 2.2)	0.46
Fetal head circumference (cm) Missing: 12	35 (± 1.6)	35 (± 1.7)	0.98
At birth			
^a^Apgar score < 7 at 5 min after birth Yes (%)	0 (0.0)	12 (0.9)	0.59
^a^PH < 7.10 (%)	3 (3.9)	54 (4.9)	0.68
^a^IUFD (%)	0 (0.0)	2 (0.1)	0.70
^a^Need for resuscitation Yes (%)	0 (0.0)	2 (0.1)	0.70
^a^Transfer to NICU Yes (%)	6 (6.5)	71 (5.3)	0.63
Composite outcomes (%)	9 (9.7)	126 (9.4)	0.93

*Abbreviations*: *IUFD* intrauterine fetal death, *NICU* neonatal intensive care unit

^*^Considered statistically significant if *p* < 0.05

^a^composite adverse neonatal outcomes: 1) pH less than 7.10 in the umbilical artery; 2) 5-min Apgar score less than 7; 3) the need for resuscitation (heart massage and /or intubation) resulting from fetal distress; 4) admission to the neonatal intensive care unit for more than 24 h; 5) IUFD; or 6) all of these. Some of the neonates who were classified as having adverse neonatal outcomes had more than one of the factors identified

A binary logistic regression was performed, and Table 5 presents the association between the risk factors and adverse composite fetal outcomes. The risk of an adverse fetal outcome was increased 2.5 times among primiparous women with RFM (AOR, 2.5, 95% CI: 1.6–3.7). Being preterm (before 37 weeks) increased the risk of an adverse outcome 5.8 times (AOR = 5.8, 95% CI: 3.2–10.4). COVID-19 infection did not increase the risk of adverse fetal outcomes (OR 1.0 (95% CI: 0.5–2.1). Multiple attendances for RFM were not associated with adverse fetal outcomes.

**Table 5:** Logistic regression of association between risk factors and adverse composite fetal outcomes for 2020. The odds ratio (OR) is presented as OR (95% confidence interval)

Risk factors for adverse composite fetal outcomes for 2020	Composite adverse fetal outcomes/total (%) 135/1,432	OR unadjusted (95% CI)	OR adjusted (95% CI)
Maternal			
Age (years)			
< 35	86/972 (8.8)	Ref	
= > 35	49/460 (10.7)	1.2 (0.8 to 1.7)	
< 40	124/1,324 (9.4)	Ref	
> = 40	11/108 (10.2)	1.1 (0.6 to 2.1)	
BMI (kg/m2)			
< 30	116/1,189 (9.8)	Ref	
= > 30	14/159 (8.8)	0.8 (0.8 to 1.5)	
Missing: 5/84			
BMI < 35	128/1,306 (9.8)	Ref	
BMI > = 35	2/42 (4.8)	0.4 (0.1–1.9)	
Parity			
Multipara	37/644 (5.7)	Ref	Ref
Primipara	98/788 (12.4)	2.3 (1.5 to 3.5)^*^	2.5 (1.6 to 3.7)^*^
SmokingA			
No	112/1,190 (9.4)	Ref	
Yes	17/144 (11.8)	1.2 (0.7 to 2.2)	
Missing: 6/98			
Chronic illnessB			
No	115/1,196 (9.6)	Ref	
Yes	16/176 (9.1)	0.9 (0.5 to 1.6)	
Missing: 4/60			
Pregnancy-related illnessC			
No	116/1,277 (9.1)	Ref	
Yes	19/155 (12.3)	1.3 (0.8 to 2.3)	
COVID-19 infectionD			
No	126/1,339 (9.4)	Ref	
Yes	9/93 (9.7)	1.0 (0.5 to 2.1)	
More than one visit for RFM			
No	103/1,162 (8.9)	Ref	
Yes	32/270 (11.9)	1.4 (0.9–2.1)	
Fetal			
Gestational age (weeks)			
> = 37	112/1,341 (8.4)	Ref	Ref^*^
< 37	20/59 (33.9)	5.6 (3.1–10.0)^*^	5.8 (3.2–10.4)
< 41	96/1,045 (9.2)	Ref	
= > 41	36/355 (10.1)	1.1 (0.7 to 1.6)	
Missing: 3/32			
SGA			
No	129/1,396 (9.2)	Ref	
Yes	6/36 (16.7)	1.9 (0.8 to 4.8)	
LGA			
No	128/1,379 (9.3)	Ref	
Yes	7/53 (13.2)	1.5 (0.7–3.4)	

A: Smoking before three months of pregnancy. B: Diabetes mellitus, cardiovascular disease, hypertension, lung disease/asthma or chronic kidney disease. C: Preeclampsia, hypertension, gestational diabetes, hepatopulmonary syndrome or gestational diabetes. D: Tested on arrival at the clinic before labour and/or when seeking care for RFM

*Abbreviations*: *RFM* reduced fetal movements, *SGA* small for gestational age, *LGA* large for gestational age

^*^Considered significant if *p* < .05. (*n* = 135/*n* = 1,432)

## Discussion

Soder Hospital in Stockholm is one of Sweden’s largest women’s clinics, with approximately 8,000 births per year and 2,000 women seeking care yearly due to RFM during pregnancy. This figure has remained constant in recent years and did not change significantly during the pandemic. The results of this study showed that fetal outcomes in the RFM group remained unchanged when the first year of the pandemic (2020) was compared with the year before (2019). There were no significant differences in fetal well-being between the COVID-19-positive and COVID-19-negative cohorts. These results indicate that fetuses from RFM pregnancies were not more likely to experience adverse outcomes at birth during the first year of the COVID-19 pandemic at the clinic under study.

We must remember that there was a difference in the management of COVID-19 in Sweden compared with other countries. There were no lockdowns, which may have affected the findings in this study. Some other countries reported an increase in stillbirths during the pandemic; the hypothesis was that the increase may be due to infection. Our results indicate this was not the case and that the increase seen in those countries is more likely associated with disruption of antenatal care provision, i.e., fewer face-to-face visits, more often telephone consults, etc., rather than COVID-19 infection. We must consider this when we compare our results with those of others.

The incidence of Swedish stillbirths has witnessed slight variation in the past 30 years, with a reported frequency of 400–450 stillbirths/year out of 115,000 births. During the pandemic, the stillbirth rate was reported as unchanged. These findings are consistent with the results of previous studies, such as those of Huntley et al., which found that COVID-19 infection was not associated with an increased risk of IUFD [19]. Furthermore, Pasternak [18] reported that the proportion of IUFDs remained unchanged during the pandemic compared to pre-COVID-19 levels. However, Chiewalska et al. reported an increase in the stillbirth rate during the COVID-19 pandemic [14]. Our results suggest that the pandemic did not harm fetal outcomes in the group with RFM. Overall, our results indicate that COVID-19 infections and pandemic measures had minimal if any, effect on fetal outcomes in our study population.

### RFM

Maternal perceptions of RFM play an essential role in attempts to identify potential adverse fetal outcomes and the risk of IUFD. Therefore, women seeking care due to RFM constitute a risk group where IUFD can be prevented. One risk identified during the pandemic was that the women were reluctant to seek care in hospitals due to the fear of being infected with the virus. Our study concluded that there was a tendency for reduced visits (19.2 vs 18.6%) for RFM in the clinic during the first wave of the pandemic (2020). However, this result was not statistically significant (*p* = 0.057).

Whether there is a pandemic or not, it is important to differentiate patients at risk of poor fetal outcomes from the heterogeneous group of pregnancies with RFM. It is well known that most pregnancies and deliveries are uncomplicated, even if the woman experiences RFM [7]. An accurate risk assessment for this group could help balance unwanted interventions/over-investigation and maintain good neonatal outcomes.

### Preterm birth

The previous literature lacks studies associating RFM with preterm birth [20]. However, our study has shown that women who experienced RFM during the pandemic gave birth to preterm babies significantly more often. The alternative was associated with an increased risk of delivering infants with adverse fetal outcomes. This study also reported a high induction rate, implying an association between sick mothers and iatrogenic preterm births due to precaution and maternal indications (COVID-19). This is in line with previous findings showing that preterm births have been associated with maternal COVID-19 infection [2].

Does COVID-19 infection pose fetal risks in RFM pregnancies?

COVID-19 has significantly impacted global health and has caused concern among pregnant women and their healthcare providers about the potential risks to maternal and fetal health. However, this study suggests an infection with COVID-19 is not a significant risk factor for adverse fetal outcomes among women presenting with RFM, a finding consistent with Stephansson et al. [21]. Numerous other studies, such as those by Huntley and Salerno [17, 19], have reported no increased risk of IUFD in pregnant women with COVID-19 infection.

This study explains why COVID-19 may not have been a significant risk factor for adverse fetal outcomes. One possibility is that the level of transmission of the virus to the fetus was low. The extent of transmission is a subject of controversy and seems to be rare [2]. A study conducted on the placental pathology of women infected with SARS-CoV-2 during pregnancy showed transmission to the placenta, which can cause SARS-CoV-2 placentitis [10]. SARS-CoV-2 placentitis leads to the destruction of the placenta, leading to placental insufficiency. This, in turn, can cause RFM and, by extension, fetal deaths due to intrauterine hypoxia. These findings suggest that placental insufficiency, rather than the direct impact of the COVID-19 infection, may be responsible for fetal fatalities [10]. This potentially explains why there was no increase in adverse fetal outcomes in our study, especially if a proportion of the women included in the study had a mild form of COVID-19 infection that did not transmit to the placenta.

### Strength and limitations

The maternal subjective perception of RFM is always tricky to measure. This did not change during the pandemic. One strength of this study is that the women attending Soder Hospital were encouraged to seek care due to RFM, regardless of whether COVID-19 infection was ongoing. The Swedish COVID-19 mitigation strategy differed from that of other countries. Sweden had no lockdown measures, and pregnant women could move around in society, albeit with some caution. This means that women who experienced RFM were encouraged to attend the hospital for a check-up, and particular hospital premises were used to securely help them with RFM, even if they were infected with COVID-19.

Another strength of this study is the large sample size (1,432 women). Further, the data were dependable and robust, as they were retrieved directly from the women’s birth records and were not acquired as register data.

Nevertheless, a significant limitation is that despite the adequate sample size, only 93 participants (6.5%) had COVID-19 infection out of 1,432. Given that there was no universal screening, the number of women with COVID-19 might have been more significant, and there might have been asymptomatic women.

Furthermore, this study did not analyse the severity of the COVID-19 infection or the time of the maternal infection. It is possible that our population had milder COVID-19 infections at the time of RFM compared to the women included in other studies, which could explain why not as many adverse fetal outcomes were observed in our study.

However, another significant limitation of the study is that it was conducted in Stockholm. This may not represent the Swedish population due to baseline factors associated with living in a big city, such as higher maternal age and educational level. Also, other socioeconomic factors related to the big city may have impacted the results of this study, potentially limiting the generalizability of the findings to different populations.

## Conclusion

This study found no increase in the proportion of adverse composite outcomes in RFM pregnancies during the first year of the pandemic. Maternal COVID-19 infection did not increase the risk of adverse composite outcomes. However, the study found that primiparity was associated with an increased risk of adverse composite outcomes.

## Data Availability

These shall be shown on request. The complete database is private, but it shall be available on request from the corresponding author following permission from the Karolinska Institute.

## Abbreviations

AOR: Adjusted odds ratio
BMI: Body mass index
CI: Confidence interval
CTG: Cardiotocography
IVF: In vitro fertilisation
IUFD: Intrauterine fetal death
NICU: Neonatal intensive care unit
OR: Odds ratio
RFM: Reduced fetal movements
